# Diagnostic application of PIK3CA mutation analysis in Chinese esophageal cancer patients

**DOI:** 10.1186/s13000-014-0153-4

**Published:** 2014-08-09

**Authors:** Zizhen Ming, Dongxian Jiang, Qin Hu, Xiaojing Li, Jie Huang, Yifan Xu, Yalan Liu, Chen Xu, Xiuguo Hua, Yingyong Hou

**Affiliations:** Shanghai Key Laboratory of Veterinary Biotechnology, School of Agriculture and Biology, Shanghai Jiao Tong University, Shanghai, 200240 China; Department of Pathology, Zhongshan Hospital, Fudan University, Shanghai, 200032 P.R. China

**Keywords:** Esophageal carcinoma, Mutation, *PIK3CA* gene

## Abstract

**Background:**

The *PIK3CA* gene mutation was found to associate with prognosis and might affect molecular targeted therapy in esophageal carcinoma (EC). The aim of this study is to compare different methods for analyzing the *PIK3CA* gene mutation in EC.

**Methods:**

Genomic DNA was extracted from 106 surgically resected EC patient tissues. The *PIK3CA* mutation status (exons 9 and 20) were screened by mutant-enrich liquid chip (ME-Liquidchip), Sanger sequencing, and pyrosequencing. And all samples with mutations were independently reassessed using amplification refractory mutation system (ARMS) methods again.

**Results:**

*PIK3CA* mutation rates were identified as 11.3% (12/106) by ME-Liquidchip. 10 mutations occurred in exon 9 and 2 in exon 20, including G1624A:E542K (n = 4), G1633A:E545K (n = 6) and A3140G:H1047R (n = 2). The results were further verified by ARMS methods. Among these 12 cases characterized for *PIK3CA* mutation, however, only 7 and 6 cases were identified by Sanger sequencing (6.6%,7/106) and pyrosequencing (5.7%,6/106), respectively.

**Conclusion:**

Sanger sequencing and pyrosequencing are less sensitive and are not efficiently applicable to the detection of *PIK3CA* mutation in EC samples. Choosing between ME-Liquidchip and ARMS will depend on laboratory facilities and expertise.

**Virtual Slides:**

The virtual slide(s) for this article can be found here: http://www.diagnosticpathology.diagnomx.eu/vs/13000_2014_153

## Background

The phosphatidylinositol 3-kinase (PI3K) signaling pathway has long been suggested to play a pivotal role in the growth and survival of the cell, and pathway activation is frequently found in the oncogenesis of human cancers [1]. High frequencies of somatic mutations conferring oncogenic potential have been found in the *PIK3CA* gene, which encodes the PI3K catalytic subunit p110α [2–7]. More than 30% of various solid tumor types were found to contain the mutations, and it was frequently mutated in cancers of the colon, breast, brain, liver, stomach and lung [3,4,8]. Mutations in the three mutation hotspots in *PIK3CA* (G1624A:E542K, G1633A:E545K, and A3140G:H1047R) have been shown to elevate the lipid kinase activity and thereby leading to the activation of the downstream Akt signaling pathway [7]. Targeted therapies such as Imatinib mesylate (anti-BCR/ABL and c-kit), Gefitinib and Erlotinib (anti-*EGFR*) that appear to impart a high degree of specificity for translocated/mutated oncogenesis give hope that therapies targeted specifically against mutant *PIK3CA* can be developed [5,9–12]. A recent report extends previous studies suggesting that the acquisition of *PIK3CA* mutations may be an important molecular event in the etiology of esophageal cancer (EC) and the mutations are associated with their clinical outcome [13–16].

The frequency of *PIK3CA* mutation in EC varied from 0% to 21%, which could likely introduce some bias in the statistical analyses of their clinical significance [6,15–18]. The difference might be due to a difference in the patient cohorts or the methods used to assess *PIK3CA* mutation. What’s more, somatic mutations of *PIK3CA* in EC samples are less than that observed in colorectal (18.8-32.0%) and breast (8.3-40%) tumors [2,8,19]. Therefore, standard methods with high sensitivity and speciality will be conductive to examine the predictive and prognostic significance of *PIK3CA* mutations in EC.

The first method used for mutation detection was direct Sanger sequencing of PCR products. However, it has been limited in its ability to identify low levels of mutation-bearing tumor cells [20,21]. The development on new molecular techniques and the increased knowledge of the genes implicated in the therapy response has boosted the development of several new detection technology including pyrosequencing, polymerase chain reaction-restricted fragment length polymorphism (PCR-RELP), Scorpion amplification refractory mutation system (Scorpion-ARMS), length analysis of fluorescently labeled polymerse chain reaction (PCR), denaturing high-performance liquid chromatography (DHPLC), and mutant-enrich liquid chip (ME-Liquidchip). However, uncertainty exists regarding which detection method can be applied in a reproductive, sensitive, and simple manner in the routine diagnostic setting. In previous studies, researchers compared the detection rate of *PIK3CA*, *EGFR*, *KRAS* and *BRAF* mutation in colorectal or lung cancers by different methods [22–25]. To our knowledge, no comparative assessment of these novel technologies has been available in EC up until now.

In this study, we compare four different methods of *PIK3CA* mutation ananlysis: Sanger sequencing, pyrosequencing, ME-Liquidchip and ARMS.

## Methods

### Patients and tissue samples

106 patients with esophageal cancer were recruited from March to October 2010 with the assistance of the Department of Thorax Surgery, Zhongshan Hospital. All samples without any treatment preoperatively were obtained intraoperatively during esophagetomy resection at our Hospital. Prior written informed consent was provided from all patients and the study protocol received ethics board approval at the Zhongshan hospital, Fudan University.

Tissues were fixed in 10% buffered formalin within 30 minutes after resection and processed following the routine procedure after 24 hours fixation. Sections were stained with hematoxylin and eosin (H&E) and reviewed by two pathologists to confirm the EC diagnosis. Then, the following patient characteristics were collected for the 106 EC patients, including gender, age, tumor site, histological grade, depth of infiltration lymph node metastasis, and stage [26].

### Genomic DNA extraction from formalin-fixed paraffin-embedded (FFPE) tissue

FFPE tumor blocks were reviewed for quality and tumor content. A single representative block, containing at least 70% of neoplastic cells, was selected for DNA extraction. Genomic DNA was extracted using the QIAamp Mini kit (Qiagen) according to the manufacturer’s instructions. The extracted DNA was stocked at −20°C for posterior analysis.

### Sanger sequencing

DNA fragments corresponding to exon 9 and 20 of the *PIK3CA* gene were amplified by PCR. The primers for exon 9 of *PIK3CA* gene were GTCTTAGATTGGTTCTTTCCTG for forward and GCATT TAATGTGCCAACTACC for reverse, for exon 20 of *PIK3CA* gene were TTTGTCTACGAAAGCCTCTCTA for forward and CCATCACTTTTTCCTTCTCCAT for reverse. The PCR products were purified using the PCR purification kit (Axygen) and checked on 2% agarose gel electrophoresis. Sequencing was performed by applying the same forward and reverse primers and using the Big Dye ®terminator sequencing kit version 3.0 (Life Technologies) on a 3130 genetic analyzer (Life Technologies). The sequencing results were interpreted using Chromas software.

### Pyrosequencing

PCR was carried out with Takara hotstart Taq. The PCR products were then prepared with the Vacuum Prep workstation (Biotage AB, Uppsala, Sweden) according to the following protocol: 40 μl of the amplicon, 3 μl streptavidin Sepharose HP beads (GE Healthcare), 37 μl binding buffer and 15 μl Milli-Q water were mixed for 5–10 min. The biotinylated amplicons were immobilized onto the streptavidin Sepharose beads, washed by 70% ethnol, denatured by 0.2 mol/l NaOH, and washed by water using the Vacuum Prep workstation. The amplicons were transferred to a plate containing 0.4 μmol/l corresponding sequencing primer in 40 μl annealing buffer. For exon 9, the sequencing primer is *PIK3CA*-9-S (AAGCAATTTCTACACGAG); for exon 20, the sequencing primer is *PIK3CA*-20-S (GGCTTTGGAGTATTTCAT). Heat the plate with the samples at 80°C for 2 min. Pyrosequencing was performed using the PyroMark Gold Q96 Reagent and the PyroMark ID system (QIAGEN). The results were compared with the sequences of human PIK3CA gene in National Center for Biotechnology Information. All mutations were verified at least twice, as were the normal controls.

### Mutant-enriched liquidchip technology (ME-Liquidchip)

The ME-Liquidchip method includes three major steps: 1) introduction of a restriction site to the wild-type or mutant DNA through PCR amplification to eliminate the wild-type genes by restriction enzyme digestion; 2) selective amplification of the mutated DNA sequence; 3) hybridization of the mutated PCR product to a specific probe, which is precoated on the polystyrene microspheres and analyzed using the Luminex (xMAP) analyzer (Luminex Co.) In this method, the wild-type DNA could not be completely digested after the first round of PCR. However, after digestion, the ratio of mutant to wild-type DNA was dramatically increased. Therefore, the mutant PCR product after the 2nd PCR could be detected using the Luminex analyzer. Primers were designed against the sequences of codon 542, 545 and 1047 in the *PIK3CA* gene. Following PCR amplification, 3 uL of PCR product was transferred to 96-well plates for Hybridization, which contained 37 uL of the hybridization solution (SurExam, China) and a mixture of 1000 probe-coated beads of each type of mutation (H1047L, H1047R, E542K, E545K, E545D). The hybridized beads were analyzed using a Luminex 200 analyzer and the median reporter fluorescence intensity (MFI) was computed for each bead set in the samples. Screening was performed by SurExam Bio-Tech Co. Ltd. (Guangzhou Technology Innovation Base, Science City, Guangzhou, P.R China).

### ARMS analysis of samples with *PIK3CA* mutation detected by other methods

DNA samples from the same preparation for PCR direct sequencing analysis were subjected to *PIK3CA* mutant analysis using AmoyDx (r) PIK3CA Five Mutations Detection Kit (Amoy Diagnostics Co.Ltd). The kit contains primers designed for detection of 5 common *PIK3CA* mutations based on ARMS PCR (amplification refractory mutation specific PCR) and double circular probe detection technology. The analysis was carried out as described in the operation instruction of the kit.

### Statistical analysis

The agreement for genotype *PIK3CA* mutational was measured by the index kappa [27] calculated using SPSS software version 15, and Statistical differences were analyzed by the McNemar test [28] and *p* value less than 0.05 was recorded as significant.

## Results

### Characterization of EC patients

A total of 106 EC samples obtained from primary tumors. The age of the patients ranged from 37 to 81 years with a median of 62 years of age. There were 88 males and 18 females. There were 1 tumor in upper, 37 in middle and 68 in lower sites. Histologically, 96 cases were squamous cell carcinoma (SCC), 2 cases were adenosquamous cell carcinoma (ASCC), 2 were basoid squamous cell carcinoma (BSCC), 3 cases were neuroendocrine carcinoma (NEC), 3 cases were composition of SCC and NEC. No pure adenocarcinoma was identified in our collection. Based on cell differentiation, 61 were graded as II, 30 were III and 5 were IV, the other cases were non-ESCC. Six cases in stage IB, 31 in stage IIA, 25 in stage IIB, 22 in stage IIIA, 19 in stage IIIB, and 3 in stage IIIC.

### Gene mutation analysis

**Figure 1:**
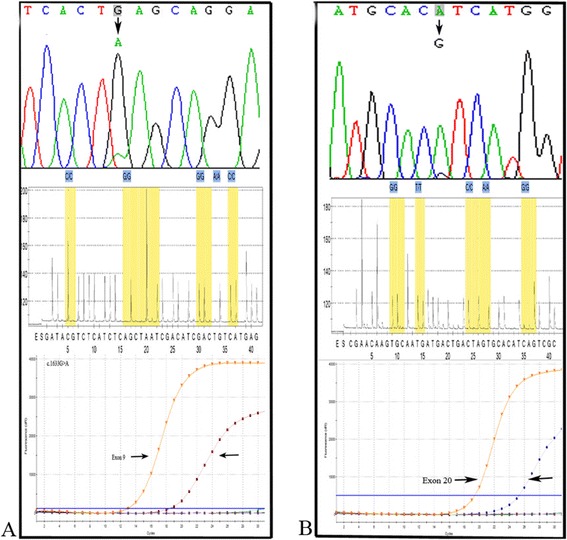
**Representative results for** ***PIK3CA*** **exon 9 and 20 mutation analysis.** EC058 **(A)** and EC037 **(B)** were detected harboring E545K and H1047R mutation using Sanger sequencing and ARMS, but the same samples were considered wt according to pyrosequencing.

*PIK3CA* mutations on exons 9 and 20 were analyzed in 106 esophageal carcinoma patients by Sanger sequencing, pyrosequencing, ME-Liquidchip. All the mutant samples detected were further assessed using ARMS. The examples of date adquisition by the different methods are shown in Figure 1. *PIK3CA* mutations rates were 5.7% (6/106), 6.6% (7/106) and 11.3% (12/106) by Sanger sequencing, pyrosequencing, and ME-Liquidchip respectively. The subset of 12 samples displaying mutant sequences were detected by ARMS, and the results were consistent with ME-Liquidchip method. The results are shown in Table 1. *PIK3CA* mutation spectrum was similar in all 4 approaches with the exception that exon 20 mutation (representing approximately 16.7% of the mutations according to ME-Liquidchip and ARMS) were not detected by pyrosequencing. We used different statistical approaches to compare methods (Tables 2 and 3). Overall, concordance between Sanger sequencing and pyrosequencing was high, but ME-Liquidchip did not correlate well with any of the alternative methods. The later was basically due to the fact that 5–6 samples displaying wildtype sequences appear to be mutated by ME-liquidchip and ARMS. The detection percentage of *PIK3CA* mutation by ME-Liquidchip was higher than that of Sanger sequencing and pyrosequencing, this difference was statistically significant (*p* < 0.05, respectively).

**Table 1:** ***PIK3CA*** **mutation status determined by different methods**

**Methods**	**ME**-**Liquidchip**		**ARMS**		**Sanger sequencing**		**Pyrosequencing**	
**Case no**	**Nucleotide**	**Amino acid**	**Nucleotide**	**Amino acid**	**Nucleotide**	**Amino acid**	**Nucleotide**	**Amino acid**
EC011	G1624A	E542K	G1624A	E542K	G1624A	E542K	G1624A	E542K
EC030	G1633A	E545K	G1633A	E545K	G1633A	E545K	G1633A	E545K
EC037	A3140G	H1047R	A3140G	H1047R	A3140G	H1047R	WT	unchanged
EC039	G1624A	E542K	G1624A	E542K	G1624A	E542K	G1624A	E542K
EC041	G1624A	E542K	G1624A	E542K	G1624A	E542K	G1624A	E542K
EC053	G1624A	E542K	G1624A	E542K	WT	unchanged	WT	unchanged
EC058	G1633A	E545K	G1633A	E545K	G1633A	E545K	WT	unchanged
EC060	G1633A	E545K	G1633A	E545K	G1633A	E545K	G1633A	E545K
EC070	A3140G	H1047R	A3140G	H1047R	WT	unchanged	WT	unchanged
EC074	G1633A	E545K	G1633A	E545K	WT	unchanged	WT	unchanged
EC084	G1633A	E545K	G1633A	E545K	WT	unchanged	WT	unchanged
EC095	G1633A	E545K	G1633A	E545K	WT	unchanged	G1633A	E545K

**Table 2:** **Comparison of mutation frequencies of the PIK3CA oncogene detected by three testing methods**

**ME**-**Liquidchip vs Sanger sequencing**				**ME**-**Liquidchip vs Pyrosequencing**				**Sanger sequencing vs Pyrosequencing**			
ME-Liquidchip				ME-Liquidchip				Sanger sequencing			
Sanger sequencing	WT	Mut	Total	Pyrosequencing	WT	Mut	Total	Pyrosequencing	WT	Mut	Total
WT	94	5	99	WT	94	6	100	WT	99	1	100
Mut	0	7	7	Mut	0	6	6	Mut	0	6	6
Total	94	12	106	Total	94	12	106	Total	99	7	106

The McNemar Test, ME-Liquidchip vs Sanger sequencing, ME-Liquidchip vs Prosequencing, Sanger sequencing vs Prosequencing were, respectively, 5.25 (p < 0.025), 6.36 (p < 0.025) and 1.01 (p > 0.25).

Wt, wild type; Mut, mutant.

**Table 3:** **Concordance between the three methods used**

**Kappa (95% CI)**	**ME-Liquidchip**	**Sanger sequencing**	**Pyrosequencing**
ME-Liquidchip		0.71 (0.59-0.83)	0.64 (0.51-0.77)
Sanger sequencing			0.75 (0.61-0.89)

The results of mutational analysis by Sanger sequencing were in agreement with Pyrosequencing k = 0.75. The scores of both methods were in poor concordance with ME-Liquidchip k = 0.71 vs to Sanger sequencing and k = 0.64 vs to Prosequencing.(values in brackets are the 95% CI for the k statistic).

Three discrepancies were observed between Sanger sequencing and pyrosequencing. Two samples (EC037 and EC058) carried mutations according to Sanger sequencing, but were negative according to pyrosequencing. On the contrary, one sample (EC095) was negative according to Sanger sequencing, but carried a mutation according to pyrosequencing. These mutations were detected as well by ME-liquidchip and ARMS.

## Discussion

The knowledge of the mutational status of many genes implicated in response to the therapy (*KIT*, *EGFR*, *BRAF*, *KRAS*, *PI3K*, etc.) [29–31] is a real fact in the clinical management of the patients. By combining the copious amount of sequencing data over the past year, we find that the *PIK3CA* gene is one of the two most commonly mutated genes identified in human cancers (the other being *KRAS*) [2–4,6,8]. In most tissue types, mutations predominantly cluster within the helical and kinase domains of the PIK3CA subunit. Exon 9 mutations are located in the helical domain of p110a and are considered to abrogate inhibitory intermolecular interaction between p85 and p110. In contrast, exon 20 mutations are located near the activation loop and are considered to produce constitutive kinase activity. Many researchers suggested that these genetic alterations may be kinase activating and oncogenic in different human malignant tumors, including colon, breast cancer, endometrial, lung, and brain tumors [32–37]. Recently, Song and Lin identified *PIK3CA* mutations in EC with whole-genome sequencing, whole-exome sequencing or array comparative genomic hybridization analysis, and suggested their functional relevance in EC [13,14]. On the other hand, the use of some potent PI3K/mTOR inhibitors, even nonsteroidal anti-inflammatory drugs (NSAID) was thought associate with longer survival among patients with mutated-*PIK3CA* colorectal cancer, but not among patients with wild-type *PIK3CA* cancer [38–41]. Therefore, *PIK3CA* mutation screening would greatly improve therapeutic strategies for EC in the future.

According to the current study, the different methods used in examination provide different mutation results, such as *KRAS* and *EGFR* in the same samples [22,42]. Several methods have been applied to detection of *PIK3CA* gene mutation in EC [15,17,18,43], it is necessary to compare the assays.

Until recently, the gold standard and most widely available method for mutation detection was Sanger sequencing, which is the only approach that permits to detect all possible sequence variants present in the target sequence, but it has several drawbacks. In particular, it is time consuming and has low sensitivity (20–50%) for the detection of mutant cells in FFPE tissue. Moreover, subjective interpretation is difficult to avoid if signal/noise ratio is low. Pyrosequencing is a sequencing-by-synthesis approach based on sequential addition of dNTPs followed by release of a pyrophosphate molecule that differs from the chain termination dye of the Sanger method, and particularly efficient for investigating mutations in short sequences. So pyrosequencing assay for *PIK3CA* mutation detection is certainly useful, because most activating *PIK3CA* mutations cluster in the hotspots of exons 9 and 20. In some cohort the minimum of mutated alleles detected with pyrosequencing was 5%. The ARMS kit has many advantages, including a closed system to prevent contamination, fast turnaround time (which may be relevant in some clinical situations), user-friendly, software-assisted data interpretation that prevents subjective interpretation. ME-Liquidchip is a novel technology, which integrates the sensitive mutant enriched PCR and quantitative high-throughput liquidchip (suspension array) to detect DNA somatic mutations in *EGFR*, *KRAS*, *BRAF*, and *PIK3CA* genes from tissues or serum samples. It has been reported that ME-Liquidchip is capable of detecting as few as 20 copies of mutant alleles with a sensitivity limit of at least mutant: wild-type ratio of 0.1%.

Here, we report our study of *PIK3CA* mutations by Sanger sequencing, pyrosequencing, ME-Liquidchip and ARMS in a clinical setting. With 106 EC samples, a total of 12 *PIK3CA* mutations were identified. The prevalence and distribution of *PIK3CA* mutations found in our study by ME-Liquidchip (11.3%) were in agreement with previous data reported in esophageal cancer [16,17,43]. All mutations were independently reassessed by ARMS. ME-Liquidchip and ARMS detected five mutated cases were not found by Sanger sequencing and pyrosequencing and they detect all mutations that had been detected by the other two methods, suggesting a higher sensitivity.

As to Sanger sequencing and pyrosequencing, the detected rate of *PIK3CA* mutation was 6.6% and 5.7%, respectively. Sanger sequencing detected two tumors harboring E545K and H1047R mutation, but the same samples were considered wt according to pyrosequencing. On the contrary, a tumor harboring a E545K mutation in terms of pyrosequencing was not detected by Sanger sequencing. Because the three mutations were detected by ME-Liquidchip and ARMS, we are pretty sure that they are true false negative in Sanger sequencing or pyrosequencing. Interestingly, the mutation frequency detected by Sanger sequencing is slightly higher compared with that of pyrosequencing. In previous study, the latter was considered to be with the very high sensitivity [15,24,42]. Most probably, they reflect problems related to low tumor DNA enrichment in the sample or the predesigned kit.

On cost-effectiveness grounds, Sanger sequencing is the method with the better defined track record and is the least expensive, relying on instruments and cheap reagents that are widely available. Pyrosequencing, ME-Liquidchip and ARMS require expensive reagents and consumables along with purchase or lease of specific instruments required by the different kits. The cost-effectiveness ratio of molecular diagnostic tests depends on a number of factors that can vary considerably, depending on the characteristics of each molecular diagnostic laboratory or the demands of specific institutions. It is important to point out that we have performed our analysis in a consecutive series of 106 EC tumor samples, corresponding to the everyday practice of most molecular diagnostic laboratories.

It’s reported there was an different distribution of *PIK3CA* mutations between adenocarcinoma and SCC histological subtypes of EC [16,43]. However, all our detected mutant samples were esophageal SCC. Striking variation in geographical distribution may be the reason. It’s known to us, esophageal SCC is the predominant histological type in Asian areas, in contrast, esophageal adenocarcinoma is the predominant histological in Western countries [44]. Our study is retrospective, the results still need to be further confirmed by more investigation.

## Conclusion

To conclude, this is so far the only study comparing these four molecular methods in EC. We have demonstrated that ME-Liquidchip and ARMS is highly sensitive for clinical diagnosis of *PIK3CA* mutation in EC. Using these methods, we found significant statistical numbers of mutated cases that were not detected by Sanger sequencing and pyrosequencing, and these results may contribute to therapeutic decision of EC.

## References

[1] Yuan TL, Cantley LC (2008). PI3K pathway alterations in cancer: variations on a theme. Oncogene.

[2] Samuels Y, Wang Z, Bardelli A, Silliman N, Ptak J, Szabo S, Yan H, Gazdar A, Powell SM, Riggins GJ, Willson JK, Markowitz S, Kinzler KW, Vogelstein B, Velculescu VE (2004). High frequency of mutations of the PIK3CA gene in human cancers. Science.

[3] Samuels Y, Velculescu VE (2004). Oncogenic mutations of PIK3CA in human cancers. Cell Cycle.

[4] Samuels Y, Ericson K (2006). Oncogenic PI3K and its role in cancer. Curr Opin Oncol.

[5] Samuels Y, Diaz LJ, Schmidt-Kittler O, Cummins JM, Delong L, Cheong I, Rago C, Huso DL, Lengauer C, Kinzler KW, Vogelstein B, Velculescu VE (2005). Mutant PIK3CA promotes cell growth and invasion of human cancer cells. Cancer Cell.

[6] Samuels Y, Waldman T (2010). Oncogenic mutations of PIK3CA in human cancers. Curr Top Microbiol Immunol.

[7] Zhao L, Vogt PK (2008). Class I PI3K in oncogenic cellular transformation. Oncogene.

[8] Karakas B, Bachman KE, Park BH (2006). Mutation of the PIK3CA oncogene in human cancers. Br J Cancer.

[9] Wallin JJ, Edgar KA, Guan J, Berry M, Prior WW, Lee L, Lesnick JD, Lewis C, Nonomiya J, Pang J, Salphati L, Olivero AG, Sutherlin DP, O’Brien C, Spoerke JM, Patel S, Lensun L, Kassees R, Ross L, Lackner MR, Sampath D, Belvin M, Friedman LS (2011). GDC-0980 is a novel class I PI3K/mTOR kinase inhibitor with robust activity in cancer models driven by the PI3K pathway. Mol Cancer Ther.

[10] Stein RC, Waterfield MD (2000). PI3-kinase inhibition: a target for drug development?. Mol Med Today.

[11] Yuan J, Mehta PP, Yin MJ, Sun S, Zou A, Chen J, Rafidi K, Feng Z, Nickel J, Engebretsen J, Hallin J, Blasina A, Zhang E, Nguyen L, Sun M, Vogt PK, McHarg A, Cheng H, Christensen JG, Kan JL, Bagrodia S (2011). PF-04691502, a potent and selective oral inhibitor of PI3K and mTOR kinases with antitumor activity. Mol Cancer Ther.

[12] Brachmann SM, Hofmann I, Schnell C, Fritsch C, Wee S, Lane H, Wang S, Garcia-Echeverria C, Maira SM (2009). Specific apoptosis induction by the dual PI3K/mTor inhibitor NVP-BEZ235 in HER2 amplified and PIK3CA mutant breast cancer cells. Proc Natl Acad Sci U S A.

[13] Song Y, Li L, Ou Y, Gao Z, Li E, Li X, Zhang W, Wang J, Xu L, Zhou Y, Ma X, Liu L, Zhao Z, Huang X, Fan J, Dong L, Chen G, Ma L, Yang J, Chen L, He M, Li M, Zhuang X, Huang K, Qiu K, Yin G, Guo G, Feng Q, Chen P, Wu Z (2014). Identification of genomic alterations in oesophageal squamous cell cancer. Nature.

[14] Lin DC, Hao JJ, Nagata Y, Xu L, Shang L, Meng X, Sato Y, Okuno Y, Varela AM, Ding LW, Garg M, Liu LZ, Yang H, Yin D, Shi ZZ, Jiang YY, Gu WY, Gong T, Zhang Y, Xu X, Kalid O, Shacham S, Ogawa S, Wang MR, Koeffler HP (2014). Genomic and molecular characterization of esophageal squamous cell carcinoma. Nat Genet.

[15] Shigaki H, Baba Y, Watanabe M, Murata A, Ishimoto T, Iwatsuki M, Iwagami S, Nosho K, Baba H (2013). PIK3CA mutation is associated with a favorable prognosis among patients with curatively resected esophageal squamous cell carcinoma. Clin Cancer Res.

[16] Phillips WA, Russell SE, Ciavarella ML, Choong DY, Montgomery KG, Smith K, Pearson RB, Thomas RJ, Campbell IG (2006). Mutation analysis of PIK3CA and PIK3CB in esophageal cancer and Barrett’s esophagus. Int J Cancer.

[17] Akagi I, Miyashita M, Makino H, Nomura T, Hagiwara N, Takahashi K, Cho K, Mishima T, Ishibashi O, Ushijima T, Takizawa T, Tajiri T (2009). Overexpression of PIK3CA is associated with lymph node metastasis in esophageal squamous cell carcinoma. Int J Oncol.

[18] Hou J, Jiang DX, Zhang JC, Gavine PR, Xu ST, Liu YL, Xu C, Huang J, Tan YS, Wang H, Lu YC, Zheng L, Hou YY, Tan LJ (2014). Frequency, characterization, and prognostic analysis of PIK3CA gene mutations in Chinese esophageal squamous cell carcinoma. Hum Pathol.

[19] Campbell IG, Russell SE, Choong DY, Montgomery KG, Ciavarella ML, Hooi CS, Cristiano BE, Pearson RB, Phillips WA (2004). Mutation of the PIK3CA gene in ovarian and breast cancer. Cancer Res.

[20] Gallegos RM, Floor K, Rijmen F, Grunberg K, Rodriguez JA, Giaccone G (2007). EGFR and K-ras mutation analysis in non-small cell lung cancer: comparison of paraffin embedded versus frozen specimens. Cell Oncol.

[21] Oliner K, Juan T, Suggs S, Wolf M, Sarosi I, Freeman DJ, Gyuris T, Baron W, Bakker A, Parker A, Patterson SD (2010). A comparability study of 5 commercial KRAS tests. Diagn Pathol.

[22] Goto K, Satouchi M, Ishii G, Nishio K, Hagiwara K, Mitsudomi T, Whiteley J, Donald E, McCormack R, Todo T (2012). An evaluation study of EGFR mutation tests utilized for non-small-cell lung cancer in the diagnostic setting. Ann Oncol.

[23] Bando I, Cillero L, Sanz-Ortega J, Llovet P, Pescador P, Ferrer M, de la Hoya M, Sastre J, Garcia ED, Caldes T (2012). Study of KRAS new predictive marker in a clinical laboratory. Clin Transl Oncol.

[24] Ihle MA, Fassunke J, Konig K, Grunewald I, Schlaak M, Kreuzberg N, Tietze L, Schildhaus HU, Buttner R, Merkelbach-Bruse S (2014). Comparison of high resolution melting analysis, pyrosequencing, next generation sequencing and immunohistochemistry to conventional Sanger sequencing for the detection of p.V600E and non-p.V600E BRAF mutations. BMC Cancer.

[25] Ney JT, Froehner S, Roesler A, Buettner R, Merkelbach-Bruse S (2012). High-resolution melting analysis as a sensitive prescreening diagnostic tool to detect KRAS, BRAF, PIK3CA, and AKT1 mutations in formalin-fixed, paraffin-embedded tissues. Arch Pathol Lab Med.

[26] Kaifi JT, Gusani NJ, Jiang Y, Mackley HB, Dye CE, Mathew A, Kimchi ET, Reed MF, Staveley-O’Carroll KF (2011). Multidisciplinary management of early and locally advanced esophageal cancer. J Clin Gastroenterol.

[27] Viera AJ, Garrett JM (2005). Understanding interobserver agreement: the kappa statistic. Fam Med.

[28] Eliasziw M, Donner A (1991). Application of the McNemar test to non-independent matched pair data. Stat Med.

[29] Di Nicolantonio F, Martini M, Molinari F, Sartore-Bianchi A, Arena S, Saletti P, De Dosso S, Mazzucchelli L, Frattini M, Siena S, Bardelli A (2008). Wild-type BRAF is required for response to panitumumab or cetuximab in metastatic colorectal cancer. J Clin Oncol.

[30] Hirsch FR, Bunn PJ (2009). EGFR testing in lung cancer is ready for prime time. Lancet Oncol.

[31] Lievre A, Bachet JB, Boige V, Cayre A, Le Corre D, Buc E, Ychou M, Bouche O, Landi B, Louvet C, Andre T, Bibeau F, Diebold MD, Rougier P, Ducreux M, Tomasic G, Emile JF, Penault-Llorca F, Laurent-Puig P (2008). KRAS mutations as an independent prognostic factor in patients with advanced colorectal cancer treated with cetuximab. J Clin Oncol.

[32] Benvenuti S, Frattini M, Arena S, Zanon C, Cappelletti V, Coradini D, Daidone MG, Pilotti S, Pierotti MA, Bardelli A (2008). PIK3CA cancer mutations display gender and tissue specificity patterns. Hum Mutat.

[33] Lee JW, Soung YH, Kim SY, Lee HW, Park WS, Nam SW, Kim SH, Lee JY, Yoo NJ, Lee SH (2005). PIK3CA gene is frequently mutated in breast carcinomas and hepatocellular carcinomas. Oncogene.

[34] Konopka B, Janiec-Jankowska A, Kwiatkowska E, Najmola U, Bidzinski M, Olszewski W, Goluda C (2011). PIK3CA mutations and amplification in endometrioid endometrial carcinomas: relation to other genetic defects and clinicopathologic status of the tumors. Hum Pathol.

[35] Pao W, Girard N (2011). New driver mutations in non-small-cell lung cancer. Lancet Oncol.

[36] Rekhtman N, Paik PK, Arcila ME, Tafe LJ, Oxnard GR, Moreira AL, Travis WD, Zakowski MF, Kris MG, Ladanyi M (2012). Clarifying the spectrum of driver oncogene mutations in biomarker-verified squamous carcinoma of lung: lack of EGFR/KRAS and presence of PIK3CA/AKT1 mutations. Clin Cancer Res.

[37] Broderick DK, Di C, Parrett TJ, Samuels YR, Cummins JM, McLendon RE, Fults DW, Velculescu VE, Bigner DD, Yan H (2004). Mutations of PIK3CA in anaplastic oligodendrogliomas, high-grade astrocytomas, and medulloblastomas. Cancer Res.

[38] Printz C (2013). Aspirin extends life of some patients with colorectal cancer. Cancer.

[39] Fang DD, Zhang CC, Gu Y, Jani JP, Cao J, Tsaparikos K, Yuan J, Thiel M, Jackson-Fisher A, Zong Q, Lappin PB, Hayashi T, Schwab RB, Wong A, John-Baptiste A, Bagrodia S, Los G, Bender S, Christensen J, Vanarsdale T (2013). Antitumor Efficacy of the Dual PI3K/mTOR Inhibitor PF-04691502 in a Human Xenograft Tumor Model Derived from Colorectal Cancer Stem Cells Harboring a Mutation. PLoS One.

[40] Kim A, Lee JE, Lee SS, Kim C, Lee SJ, Jang WS, Park S (2013). Coexistent mutations of KRAS and PIK3CA affect the efficacy of NVP-BEZ235, a dual PI3K/MTOR inhibitor, in regulating the PI3K/MTOR pathway in colorectal cancer. Int J Cancer.

[41] Din FV, Valanciute A, Houde VP, Zibrova D, Green KA, Sakamoto K, Alessi DR, Dunlop MG (2012). Aspirin inhibits mTOR signaling, activates AMP-activated protein kinase, and induces autophagy in colorectal cancer cells. Gastroenterology.

[42] Altimari A, de Biase D, De Maglio G, Gruppioni E, Capizzi E, Degiovanni A, D’Errico A, Pession A, Pizzolitto S, Fiorentino M, Tallini G (2013). 454 next generation-sequencing outperforms allele-specific PCR, Sanger sequencing, and pyrosequencing for routine KRAS mutation analysis of formalin-fixed, paraffin-embedded samples. Onco Targets Ther.

[43] Mori R, Ishiguro H, Kimura M, Mitsui A, Sasaki H, Tomoda K, Mori Y, Ogawa R, Katada T, Kawano O, Harada K, Fujii Y, Kuwabara Y (2008). PIK3CA mutation status in Japanese esophageal squamous cell carcinoma. J Surg Res.

[44] Montgomery EF, Boffetta P, Daigo Y, Shimizu M, Shimoda T (2010). WHO Classification of Tumors of the Oesophagus.

